# Incomplete immune response to coxsackie B viruses associates with early autoimmunity against insulin

**DOI:** 10.1038/srep32899

**Published:** 2016-09-08

**Authors:** Michelle P. Ashton, Anne Eugster, Denise Walther, Natalie Daehling, Stephanie Riethausen, Denise Kuehn, Karin Klingel, Andreas Beyerlein, Stephanie Zillmer, Anette-Gabriele Ziegler, Ezio Bonifacio

**Affiliations:** 1 DFG-Center for Regenerative Therapies Dresden, Dresden, Germany; 2 Institute of Diabetes Research, Helmholtz Zentrum München and Forschergruppe Diabetes, Klinikum rechts der Isar, Technische Universität München, Neuherberg, Germany; 3 Department of Molecular Pathology, University Hospital Tübingen, Tübingen, Germany; 4 Forschergruppe Diabetes e.V., Neuherberg, Germany; 5 German Center for Diabetes Research (DZD), Neuherberg, Germany

## Abstract

Viral infections are associated with autoimmunity in type 1 diabetes. Here, we asked whether this association could be explained by variations in host immune response to a putative type 1 etiological factor, namely coxsackie B viruses (CVB). Heterogeneous antibody responses were observed against CVB capsid proteins. Heterogeneity was largely defined by different binding to VP1 or VP2. Antibody responses that were anti-VP2 competent but anti-VP1 deficient were unable to neutralize CVB, and were characteristic of children who developed early insulin-targeting autoimmunity, suggesting an impaired ability to clear CVB in early childhood. In contrast, children who developed a GAD-targeting autoimmunity had robust VP1 and VP2 antibody responses to CVB. We further found that 20% of memory CD4^+^ T cells responding to the GAD65_247-266_ peptide share identical T cell receptors to T cells responding to the CVB4 p2C_30-51_ peptide, thereby providing direct evidence for the potential of molecular mimicry as a mechanism for GAD autoimmunity. Here, we highlight functional immune response differences between children who develop insulin-targeting and GAD-targeting autoimmunity, and suggest that children who lose B cell tolerance to insulin within the first years of life have a paradoxical impaired ability to mount humoral immune responses to coxsackie viruses.

Type 1 diabetes is preceded by autoimmunity^1^,^2^. Viral infections have been repeatedly reported to be associated with autoimmunity and type 1 diabetes^3^,^4^,^5^,^6^,^7^. While it is often assumed that these associations represent increased exposure to virus, they may also reflect an impaired response or alternatively, more pronounced physical symptoms during infection. Rare reports have addressed how type 1 diabetes susceptible children respond to immune challenges. We reported an impaired antibody response to tetanus vaccination in children who develop beta cell autoantibodies very early in life, and normal responses in children who developed autoantibodies at a later age^8^. Since then it has emerged that very early beta cell autoimmunity initiates against insulin, whereas beta cell autoimmunity that develops after age 3 years is usually directed against glutamic acid decarboxylase (GAD)^9^,^10^. The development of beta cell autoimmunity in these two age periods also has distinct HLA associations and different rates of progression to diabetes^9^,^10^,^11^. Therefore, our observation with respect to heterogeneous vaccination responses appears plausible, and, if generalizable to other immune challenges, would support the notion that insulin- and GAD-targeted beta cell autoimmunity can arise by distinct mechanisms reflected by aberrant and/or divergent responses to immune challenges such as infection. We reasoned that exposure to coxsackie B viruses (CVB) may be a suitable model to test this hypothesis since it has been repeatedly suggested to have an etiological role in the pathogenesis of type 1 diabetes^6^,^12^,^13^, including aberrant responses^14^,^15^. We chose the IgG antibody response as a measure of prior exposure to virus, and established immunoassays that could measure IgG antibodies to each of the four viral capsid polypeptide antigens in order to assess the heterogeneity of host response to virus exposure. Heterogeneity in the development of beta cell autoimmunity was defined by target autoimmunity (insulin, GAD, or none) age (before or after age 3 years) defined in children who were prospectively followed to beta cell autoimmunity from birth.

## Results

### The humoral response to CVB capsid proteins is heterogeneous and dominated by VP2 and/or VP1 antibodies

A panel of immunoprecipitation assays that detect the four viral capsid proteins (VP1, VP2, VP3 and VP4) of CVB1-6 was established. These assays were successful for all but CVB2 VP2 antibodies (data not shown). In an initial analysis of sera from patients with type 1 diabetes and controls, anti-VP3 antibodies for all six CVB serotypes were rare and were, therefore, not measured in the remainder of the samples (Supplementary Fig. 1).

**Figure 1:**
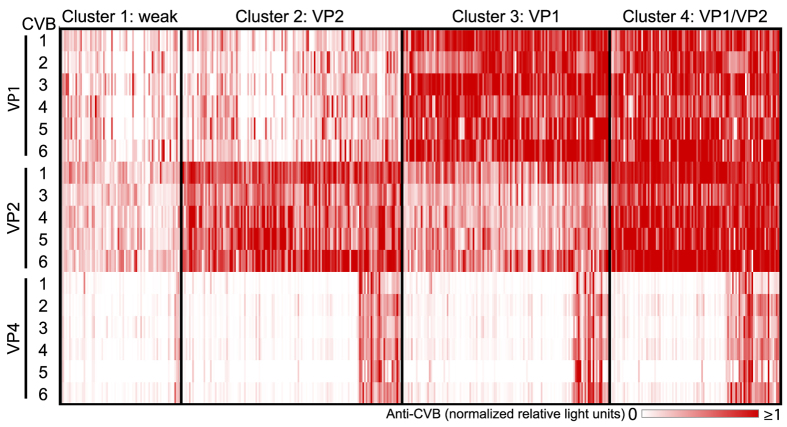
Antibody responses against CVB capsid proteins. Antibody titers against VPs of CVB1-6 are represented as a heat map where each vertical line is a sample (n = 440), and the intensity of the color corresponds to the relative light units (RLU) immunoprecipitated in the LIPS assay. Four clusters were identified by hierarchical clustering of Euclidean distances calculated from anti-VP1 and anti-VP2 antibody titers. The clusters represent samples with weak responses to VP1 and/or VP2 (cluster 1, weak), samples with strong and dominant VP2 antibody responses (cluster 2, VP2), samples with strong and dominant VP1 antibody responses (cluster 3, VP1), and samples with strong VP1 and VP2 antibody responses (cluster 4, VP1/VP2). Within each cluster, there is a small subset of samples with strong antibodies to VP4.

Anti-CVB antibody responses were heterogeneous (Fig. 1). Anti-VP2 antibodies appeared to be the most dominant, followed by anti-VP1 antibodies. Antibodies to VP4 were infrequent, which may be due to its location on the internal side of the viral capsid^16^. Increased anti-VP1 (p > 0.0001) and anti-VP4 (p = 0.048) titers were observed in adolescents as compared to children aged 3 years (Supplementary Table 1), presumably due to a higher cumulative number of exposures to the virus. Samples with no response against all capsid proteins of all 6 CVB were rare. Strong correlations were observed between CVB for each of the anti-VP antibodies (Supplementary Fig. 2), suggesting cross-reactivity of antibodies to the same antigenic region of different CVB serotypes. In contrast, little correlation was observed between anti-VP1, anti-VP2, and anti-VP4 for any of the CVB, suggesting heterogeneity in the response to the different viral capsid antigenic components. This heterogeneity was defined by four distinct signatures (Fig. 1). Cluster 1 was characterized by weak or absent antibody responses to VP1 and VP2, cluster 2 by a strong and dominant anti-VP2 antibody response, cluster 3 by a strong and dominant anti-VP1 antibody response, and cluster 4 by strong antibody responses to both VP1 and VP2.

### Heterogeneity reflects the neutralizing capacity of CVB antibodies

**Figure 2:**
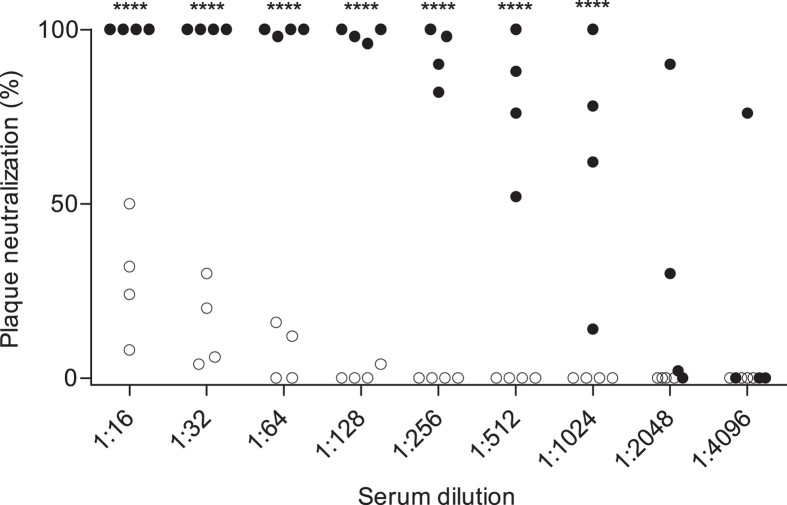
Anti-VP1 antibodies inhibit CVB plaque formation. Plaque neutralization tests using CVB3 were performed with 8 representative sera that were selected based on their anti-CVB profile (Supplementary Fig. 3) as strong anti-VP2 and VP1 antibodies (filled symbols), or strong anti-VP2 antibodies only (open symbols). Despite similar anti-VP2 titers in each serum, the anti-VP1 high titer sera had >100-fold stronger neutralization capacity than the anti-VP1 deficient sera. ****p-value < 0.0001 based on repeated measures ANOVA with Bonferroni post-test comparisons.

Humoral responses to CVB are typically measured by standard enzyme immunoassay using VP1 peptide antigen only or by neutralizing antibody assays^17^. We were intrigued, therefore, by the considerable proportion of samples with a dominant VP2 response, and asked whether the divergent anti-VP1 and anti-VP2 antibody responses were associated with differences in the ability to neutralize the virus, and thereby provide protection from infection (Fig. 2). We used CVB3 as the model virus for the neutralization antibody measurement, and chose sera with strong VP1 and VP2 antibody binding or strong VP2 only antibody binding to all six CVB (See Supplementary Fig. 3 for anti-VP1 and anti-VP2 titers). Strikingly, the samples with strong anti-VP1 and anti-VP2 antibodies strongly inhibited plaque formation with >50% inhibition at titers ≥1/512, whereas the sera with strong anti-VP2 responses, but no or weak anti-VP1 antibodies failed to reach 50% inhibition at serum titers of 1/16 (p < 0.0001). We suggest, therefore, that responses to CVBs that include anti-VP2 antibodies with weak or no anti-VP1 antibodies as seen in clusters 1 and 2 (Fig. 1) are indicative of exposure to CVB with an impaired or incomplete response, whereas the presence of moderate and strong anti-VP1 antibodies as seen in clusters 3 and 4 (Fig. 1) indicate exposure and effective antiviral response.

### Insulin autoimmunity is associated with a deficient anti-VP1 antibody response

Having demonstrated heterogeneity and an ability to distinguish exposure and response efficacy, we asked whether CVB antibodies were altered in children who developed early insulin autoantibodies or GAD autoantibodies. We expected that the majority of children would have been exposed to CVB by age 3 years, and, therefore, examined samples taken at age 3 years from children followed from birth for this purpose. Children were selected and categorized into three groups: beta cell autoantibody negative throughout follow-up (no autoimmunity); developing insulin autoantibodies as their first beta cell autoantibody prior to age 3 years (early insulin autoimmunity); and developing GAD autoantibodies as their first beta cell autoantibody (GAD autoimmunity).

**Figure 3:**
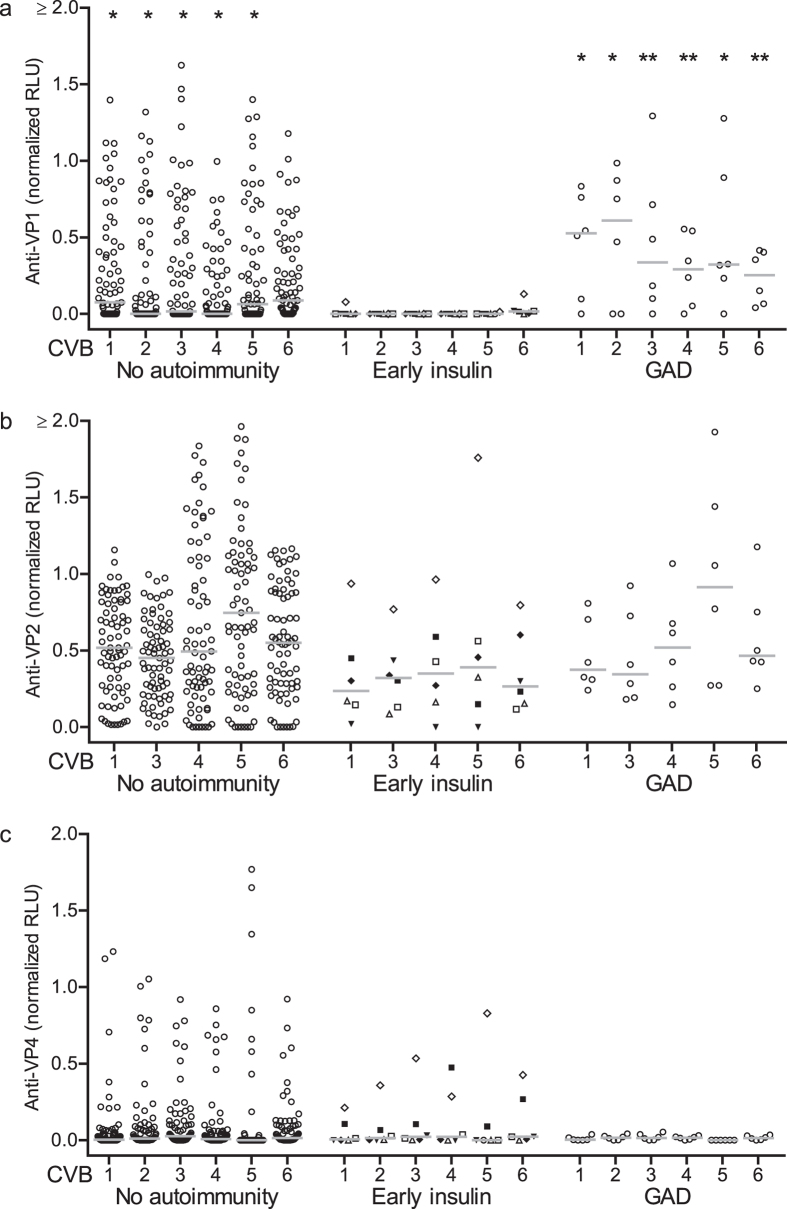
Early insulin autoimmunity is associated with a reduced anti-VP1 CVB antibody response. Anti-CVB antibodies in sera from 89 children genetically at risk for type 1 diabetes and followed for beta cell autoantibody development in the BABYDIET study. Anti-VP1 (**a**), anti-VP2 (**b**) and anti-VP4 (**c**) antibodies at 3 years of age (y axis) are shown in children stratified as beta cell autoantibody negative throughout follow-up (no autoimmunity), beta cell autoantibody seroconversion before age 3 years with IAA as the first detected autoantibodies (early insulin), and beta cell autoantibody seroconversion with GAD as the first detected autoantibody target (GAD). Each point is an individual and the horizontal bar indicates the median. For the early insulin autoimmunity children, the unique symbols correspond to data points of an individual child. *p-value < 0.05 or **p-value < 0.01 based on comparison with early insulin autoimmunity group for the corresponding CVB serotype using the Mann Whitney U test.

All children who developed early insulin autoimmunity had no or weak anti-VP1 antibody responses (Fig. 3a). Anti-VP1 antibody responses in these children were lower than the responses in children who did not develop beta cell autoimmunity (p < 0.05 for CVB 1, 2, 3, 4, 5) and children who developed GAD autoimmunity (p < 0.05 CVB1, 2, 5 and p < 0.001 CVB3, 4, 6). Anti-VP1 antibodies were also negative at the development of autoimmunity in the early insulin autoimmunity children (Supplementary Fig. 4). No differences between the autoantibody negative children and early insulin autoimmunity children or children who developed GAD autoimmunity were observed for anti-VP2 and anti-VP4 antibodies (Fig. 3b,c). Importantly, the majority of early insulin autoimmunity children had anti-VP2 or anti-VP4 antibodies at age 3 years, suggesting that they had been previously exposed to CVB (Fig. 3b,c).

### The altered anti-VP1 response is not reflected by maternally transferred anti-CVB antibodies

**Figure 4:**
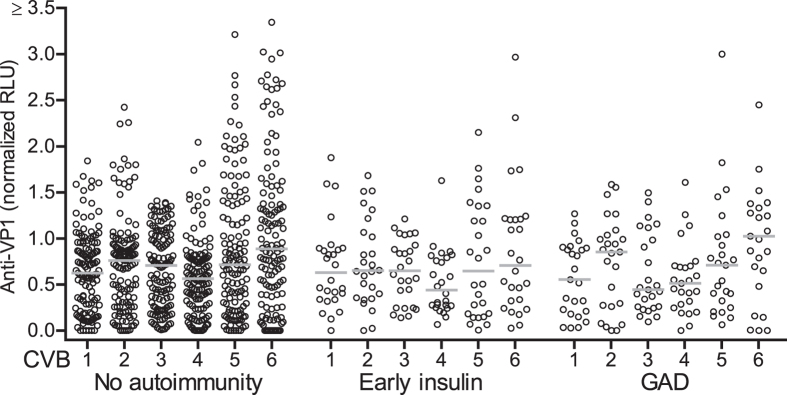
Maternal anti-CVB antibodies are not associated with beta cell autoimmunity. Anti-VP1 antibodies in cord blood serum of BABYDIAB study participants stratified as beta cell autoantibody negative throughout follow-up (no autoimmunity), beta cell autoantibody seroconversion before age 3 years with IAA as the first detected autoantibodies (early insulin), and beta cell autoantibody seroconversion with GAD as the first detected autoantibody target (GAD). Each point is an individual and the horizontal bar indicates the median. No significant differences based on comparison with early insulin autoimmunity group for the corresponding CVB serotype using the Mann Whitney U test.

It has been suggested that maternal CVB infection may increase the risk for beta cell autoimmunity in children^18^,^19^,^20^,^21^ and/or that inadequate maternal protection is a risk factor for type 1 diabetes^21^. We asked whether the low anti-VP1 antibody response observed in the children with early insulin autoimmunity could reflect a defective maternal CVB antibody status at birth. We analyzed the anti-CVB antibody titers of newborns. Robust titers of anti-VP1 antibodies were observed in the cord blood of the majority of children who developed insulin autoimmunity (Fig. 4). No differences in titers of anti-VP1, anti-VP2, or anti-VP4 CVB antibodies were observed between children who never developed beta cell autoimmunity, children who developed insulin autoimmunity, and children who developed GAD autoimmunity (Fig. 4, Supplementary Fig. 5). Moreover, the titers of maternally transferred VP1 and VP2 antibodies in cord blood were generally higher than those observed in 11–13 year old patients recently diagnosed with type 1 diabetes and healthy controls (Supplementary Fig. 6). These data suggest that children who develop early insulin autoimmunity are not deficient in their maternally transferred anti-CVB antibodies, and that the status of maternal anti-CVB antibodies does not influence anti-CVB antibody responses that develop later in life.

### CVB4 and GAD antigens are recognized by the same T-cell receptor (TCR)

It was noteworthy that children with GAD autoimmunity in our study had prominent anti-VP1 and anti-VP2 CVB antibodies. This prompted us to re-examine the CVB4/GAD65 molecular mimicry theory^14^,^15^. In order to find evidence for this, we sought to identify CD4^+^ memory T cell clones that responded to both GAD and CVB4 antigens. CD4^+^ CD45RO^+^ T cells isolated from three individuals were stimulated with monocyte-derived dendritic cells loaded with either GAD65_247-266_ peptide or CVB4 p2C_30-51_ peptide. Responsive T cells from both stimulation conditions were single-cell sorted (Supplementary Fig. 7) and TCR-α and TCR-β sequence analysis was performed. TCR sequence information was obtained for 29 CVB4 p2C_30-51_ peptide responsive and 21 GAD65_247-266_ peptide responsive cells in donor BC928, allowing us to examine overlap (Table 1). Remarkably, 4 of the 29 CVB4 p2C_30-51_ peptide responsive cells had identical TCR-α and TCR-β sequences to 4 of the 19 GAD65_247-266_ peptide responsive cells from donor BC928. These 8 cells were represented by three TCR sequences (Table 2). Insufficient sequence information was obtained from the remaining samples to allow analysis of sharing (Table 1, Supplementary Table 2).

## Discussion

We show a functionally relevant heterogeneity in the antibody response to CVB, putative etiological agents in type 1 diabetes, and demonstrate that children who developed early insulin-targeting autoimmunity had CVB response profiles associated with weak protection, whereas competent responses to CVB were observed in children who developed GAD-targeting autoimmunity. We further showed the potential for cross-reactivity between CVB and GAD at the level of the TCR. The findings are consistent with heterogeneity in beta cell autoimmunity and the mechanisms that promote autoimmunity to different beta cell autoantigens.

This is the first study to comprehensively look at antibody responses to CVB antigens in children who develop beta cell autoimmunity. Previous studies have focused on neutralizing or VP1 antibody responses and as such could not ascertain exposure when these were negative. Unexpectedly, antibodies to VP2 were as prevalent and dominant as anti-VP1 antibodies, there were responses to the internally exposed VP4 in a number of samples, and there was marked heterogeneity in the profiles against VP1, VP2, and VP4. Moreover, there was a clear association between neutralizing capacity and anti-VP1 responses, suggesting that children who mount a strong anti-VP1 response are more likely to be protected from infection. Although we have only demonstrated a relationship between anti-VP1 antibody binding and neutralization capacity for CVB3, our finding is consistent with previous data suggesting that the VP1 neutralizing capacity was linked to the BC loop on the CVB4 structural protein VP1^22^.

An ability to measure multiple target viral antigens was important and allowed us to assess our hypothesis raised by previous vaccination response observations^8^. Although we did not have a validation cohort and the number of children with early insulin autoimmunity was small, the incomplete antibody response to CVB seen in the children with early autoimmunity is entirely consistent with the findings from the tetanus toxoid antibody responses after vaccination. Thus, using two different cohorts and two models of exogenous antigen exposure, we found evidence that children who develop early insulin-targeted autoimmunity have a compromised humoral immune response in early childhood. The cause of this immune response deficiency is unclear. For enteroviruses, poor protection from infection by inadequate maternal transfer of antibodies was suggested as a potential cause for increased infections in children who develop beta cell autoimmunity^23^. However, our findings of robust anti-VP1 antibodies in cord blood of the newborns who developed either insulin or GAD targeted autoimmunity did not support this hypothesis.

Data from Finland have shown that neutralizing antibodies against CVB1 occur more often, while neutralizing antibodies against CVB3 and CVB6 occur less frequently in autoantibody positive children^6^. The authors therefore suggest that infection with specific CVB serotypes can either increase or decrease the risk of beta cell autoimmunity. Our antibody binding measurements could not show strain specific differences. Our findings also show that only measuring neutralizing or anti-VP1 antibodies to CVB, as is often performed, is insufficient to differentiate specific immune reactions against CVB. Therefore, without the measurement of anti-VP2 antibodies, it is possible that the findings in Finnish children also reflect incomplete responses to CVB3 and CVB6. Indeed, our findings raise speculation as to whether the ever-growing evidence for a role of virus infection results from an inability to competently respond to early infection. It is also possible that increased infectious events observed in children who develop type 1 diabetes are epiphenomena of a more generalized compromised immune response. It will be important, therefore, to comprehensively examine responses to other viruses using methods that provide large coverage of epitopes and viruses^24^,^25^.

Finally, our data suggests heterogeneity with respect to CVB antibody response that segregates with the target beta cell antigen. In this context, the children who initiated their beta cell autoimmunity against GAD had competent anti-VP1 and anti-VP2 responses. We, therefore, considered whether virus response mechanisms that promote autoimmunity might be operating to cause GAD autoimmunity. Molecular mimicry between the PEVKEK sequence of GAD65 and the protein 2C of CVB4 has been proposed^14^,^15^,^26^, but to date, direct evidence for cross-reactivity between these proteins has not been forthcoming. Here, we have identified identical TCRs from GAD_247-266_ and CBV4 p2C_30-51_ responding memory CD4^+^ T cells. We have not previously observed TCR identity in CD4^+^ T cells that respond to different antigens^27^,^28^, and, therefore, find the observation, albeit from a single individual, striking. Although we lack formal proof that these TCRs can specifically bind and respond to both peptides, our findings are the first evidence from the adaptive immune response that exposure to CVB4 could indeed activate GAD-specific CD4^+^ T cells via molecular mimicry.

In conclusion, we show functional immune response differences between children who develop insulin-targeting and GAD-targeting autoimmunity in childhood, and reinforce the concept that children who lose B cell tolerance to insulin within the first years of life have a paradoxical impaired ability to mount humoral immune responses to exogenous antigen exposure. The findings regarding the diversity in viral antibody titers raise the possibility that viral clearance may be impaired in children with early insulin autoimmunity.

## Methods

### Subjects

For the assessment of CVB antibody heterogeneity, sera were obtained from 440 children or adolescents. These included samples from children enrolled in the prospective German BABYDIET study (n = 89; median age, 2.9 years; interquartile range, 2.8 to 3.0 years; 54 girls)^29^, the DiMelli study (n = 78; median age, 12.2 years; interquartile range, 10.5–13.4 years; 31 girls)^30^ and TEENDIAB study (n = 78; median age, 12.2 years; interquartile range, 10.6–13.2 years; 29 girls)^31^, and cord blood serum from neonates in the BABYDIAB study (n = 195; 92 girls)^32^. The BABYDIET and BABYDIAB studies followed children with a family history of type 1 diabetes from birth. Follow-up included measurement of beta cell autoantibodies to insulin (IAA), GAD, insulin-associated antigen-2, and Zinc transporter 8 using radiobinding assays^33^,^34^,^35^. Positivity was defined as previously described^9^. The DiMelli study recruits patients diagnosed with T1D before 20 years of age (median days after diagnosis, 9; interquartile range, 6 to 12 days). Healthy control sera from the TEENDIAB study were age and sex-matched to the DiMelli sera. All sera were analyzed for CVB antibodies as blinded coded samples. The ethical committees of Bavaria or the Ludwig-Maximilians University approved the studies, which were carried out in accordance with the Declaration of Helsinki, as revised in 2000. Informed, written consent was obtained from patients or parents of participants. For isolation of antigen-specific T cells, peripheral blood mononuclear cells (PBMCs) were obtained from a healthy blood donor (BC982) of the Deutsche Rotes Kreuz Blutspendedienst Ost GmbH Dresden, an individual with recent onset T1D enrolled in the DiMelli study with sample collection at time of disease diagnosis (D161), and an individual with beta cell autoantibodies (B858).

### Generation of luciferase fusion proteins

CVB1 (GenBank# M16560.1), CVB2 (GenBank# AF081485.1, AF0852363.1), CVB3 (GenBank# M88483.1), CVB4 (GenBank# X05690.1), CVB5 (GenBank# AF114383.1) and CVB6 (GenBank# AF039205.1, AF105342.1, AF114384.1) viral RNA was generously provided by Dr. Merja Roivainen. cDNA templates of the viral capsid proteins VP1, VP2, VP3 and VP4 of all six CVB serotypes were synthesized and then amplified by PCR using primers with specific restriction sites (sequences available upon request). PCR products of the antigen DNA were inserted into the pTNT™ vector (Promega) containing either nano (Nluc), renilla (Rluc) or firefly (Fluc) luciferase, using the flanking restriction sites added and creating an in-frame fusion of the antigen with the luciferase gene. Construct integrity and accuracy was confirmed by DNA sequencing before and after subcloning. Due to the high mutation rate of enteroviruses, some divergence was observed compared with published sequences (sequence information available upon request). Fusion proteins were transcribed and translated by incubating 2 μg of plasmid DNA with the TNT^®^ Quick Coupled Transcription/Translation System (Promega) as described^36^.

### Luciferase immunoprecipitation systems assay

Luciferase activity of translated fusion proteins were determined by combining the fusion antigen with the corresponding luciferase substrate and measuring the relative light units (RLU) with a GloMax^®^-Multi Detection System (Promega). The immunoprecipitation assay was performed in a 96-well plate format by diluting the equivalent of 5 × 10^6^ RLUs of fusion protein with 1 μL of sera in TBST (20 mM Tris, pH 7.6, 1.38 M NaCl, 0.05% Tween-20) to a final volume of 35 μL. After incubation for 2 hours at room temperature, 25 uL of each sample mix was transferred to a 96-well MultiScreen_HTS_ GV Filter Plate, 0.22 μm (Merck Millipore) containing 50 μL of a 12.5% nProteinA Sepharose 4 fast flow bead suspension (GE Healthcare) and incubated for a further hour with continuous shaking at 300 rpm at 4 °C. Captured proteins were washed 10 times with 200 μl TBST using a vacuum manifold, after which all remaining liquid was removed. A light reaction was induced using the Nano-Glo^®^, Renilla or Firefly Luciferase Assay System (Promega). 25 μL of the appropriate lysis buffer was added to each well and then 25 μL of the corresponding luciferase substrate was added to each well by hand (Nluc) or by the GloMax^®^-Multi Detection System immediately prior to fluorescence detection (Rluc, Fluc). The RLU measurement of sera was corrected for background by subtracting the RLU of sera negative controls and normalized by dividing by the corrected RLU of a positive control (polyclonal rabbit antibody) that binds to all CVB antigens analyzed. Data was analyzed using KNIME version 2.5.2^37^.

### Neutralizing antibody tests

The presence of neutralizing antibodies was measured against CVB3 (Nancy strain) by using a plaque neutralization assay. Vero cells were cultured in Dulbecco’s modified Eagle’s medium (DMEM) supplemented with 10% fetal calf serum (FCS), 100 U/mL penicillin and 100 μg/mL streptomycin in 12 well plates. The cells were used at a confluence of 80–90% for the neutralization assay. Two-fold serial dilutions of the sera in DMEM (1:8 to 1:2048) were mixed with an equal volume of a virus solution containing 50 plaque-forming units. The mixtures were incubated for 1 h at 37 °C (final sera dilutions were 1:16 to 1:4096). The cells were washed with phosphate buffered saline and 300 μL of the respective serum-virus suspension were added per well. After incubation on a shaker for 1 h at room temperature the serum-virus suspension was removed, the cells were overlaid with plaque medium (1% agarose, 25 mM MgCl_2_, 3% FCS in DMEM) and incubated for 24 h at 37 °C with 5% CO_2_. After removal of the plaque medium the cells were fixed with 5% trichloroacetic acid for 45 min and stained with 5% crystal violet in 20% ethanol for 10 min. The number of viral plaques was counted and neutralization titers were defined. The neutralizing antibody measurements were performed twice on all tested sera with similar results.

### Isolation of antigen-specific CD4^+^ memory T cells

Monocyte-derived dendritic cells (mo-DCs) were obtained by culturing CD14^+^ monocytes in the presence of 50 ng/mL GM-CSF and 10 ng/mL IL-4 for 6 days. Mo-DCs were cultured with 10 μg of recombinant human Glutamic Acid Decarboxylase (GAD65, Diamyd Diagnostics) or lysate from a CVB4 infected green monkey kidney cell line (infectivity titer 1.33 × 10^9^ TCID_50_/mL) for 6 hours before adding 10 ng/mL LPS (Sigma) and 200 U/mL IFNγ (Sigma). After overnight incubation, 50 μg/mL of GAD65_247-266_ peptide (NMYAMMIARFKMFPEVKEKG) or CVB4 p2C_30-51_ peptide (WLKNKLIPEVKEKHEFLSRL) was added for three hours before peptide-loaded mo-DCs were harvested. A pure population of memory CD4^+^ T cells was obtained from PBMCs by first isolating CD4^+^ T cells using the CD4^+^ T cell Isolation Kit II (Miltenyi). CD4^+^ T cells were then labeled with CD45RO Microbeads (Miltenyi) to enrich for CD45RO^+^ T cells by magnetic activated cell sorting. CD45RO^+^ cells were labeled with CFSE (Thermo Fischer Scientific) and 150,000 cells were seeded into 96-well culture plates containing 3000 peptide-loaded mo-DCs. After 5 days incubation, cells were harvested and labeled with CD4-Pacific Blue (RPA), CD25-PE (M-A251), CD45RA-APC (HI100), CD45RO-PECy7 (UCHL1) and the live/dead cell marker 7AAD from BD Biosciences. 7AAD^−^CD4^+^ CD25^hi^CFSE^lo^CD45RO^+^ CD45RA^−^ cells were single cell sorted using the BD FACSAria^TM^ II into 96-well PCR plates containing 5 uL DEPC-PBS, which were immediately frozen on dry ice.

### T-cell receptor sequence analysis

Reverse transcription–polymerase chain reaction amplification and sequencing of TCR-α and TCR-β chains from single T cells were performed as described previously^28^. Analysis of TCR-α and TCR-β sequences and junction peptide amino acid sequence extraction was conducted with reference to the IMGT database^38^. Junction peptides were analyzed using KNIME version 2.5.2^37^.

### Statistical Analysis

Comparisons of quantitative data between groups were made by Mann Whitney U test, and categorical data using Fisher’s exact test. Neutralization antibody titers were log_2_ transformed prior to comparison. Correlations were analyzed by Pearson’s correlation coefficient. For all statistical analyses a two-tailed p value < 0.05 was considered significant. Clusters were identified by K-medoid clustering of Euclidean distances calculated from anti-VP1 and anti-VP2 antibody measurements. Analyses were performed with R version 3.3.2, GraphPad Prism 5 and KNIME version 2.5.2^37^.

## Additional Information

**How to cite this article**: Ashton, M. P. *et al.* Incomplete immune response to coxsackie B viruses associates with early autoimmunity against insulin. *Sci. Rep.* **6**, 32899; doi: 10.1038/srep32899 (2016).

## Supplementary Material

Supplementary Information

## Figures and Tables

**Table 1:** Paired TCR sequence analysis of CVB4 p2C and GAD65 responsive CD4^+^ CD45RO^+^ T cells.

Subject	Peptide stimulus	Sorted T cells (n)	T cells with paired TCR sequences (n)	T cells with TCR in both GAD and CVB4 responsive cells (n)
BC928	CVB4 p2C_30-51_	128	29	4
	GAD65_247-266_	95	21	4
B858	CVB4 p2C_30-51_	5	0	0
	GAD65_247-266_	14	7	0
D161	CVB4 p2C_30-51_	3	3	0
	GAD65_247-266_	16	0	0

**Table 2:** Paired TCR sequences shared between CVB4 p2C peptide and GAD65 peptide responsive CD4^+^ CD45RO^+^ T cells.

Antigen responsive T cells (n)		TRAV	TRAJ	JunctionA	TRBV	TRBJ	TRBD	JunctionB
CVB4 p2C_30-51_	GAD65_247-266_							
1	1	2*01	29*01	CASGNTPLVF	20-1*01	2-5*01	2*01	CRARDPGTGQETQYF
2	1	26-1*01	26*01	CIVRVSHN*G#RDNYGQNFVF	20-1*01	2-5*01	2*01	CSARDPTSGQETQYF
1	2	36/DV7*02	40*01	CAVEGLYKYIF	6-2*01	2-1*01	2*01	CASGYGDEPSLEF

## References

[b1] Todd J. A. Etiology of type 1 diabetes. Immunity 32, 457–467, doi: 10.1016/j.immuni.2010.04.001 (2010). 20412756

[b2] Ziegler A. G. & Nepom G. T. Prediction and pathogenesis in type 1 diabetes. Immunity 32, 468–478, doi: 10.1016/j.immuni.2010.03.018 (2010). 20412757 PMC2861716

[b3] Beyerlein A., Donnachie E., Jergens S. & Ziegler A. G. Infections in Early Life and Development of Type 1 Diabetes. Jama 315, 1899–1901, doi: 10.1001/jama.2016.2181 (2016). 27139064

[b4] Beyerlein A., Wehweck F., Ziegler A. G. & Pflueger M. Respiratory infections in early life and the development of islet autoimmunity in children at increased type 1 diabetes risk: evidence from the BABYDIET study. JAMA pediatrics 167, 800–807, doi: 10.1001/jamapediatrics.2013.158 (2013). 23818010

[b5] Honeyman M. C. *et al.* Association between rotavirus infection and pancreatic islet autoimmunity in children at risk of developing type 1 diabetes. Diabetes 49, 1319–1324 (2000). 10923632 10.2337/diabetes.49.8.1319

[b6] Laitinen O. H. *et al.* Coxsackievirus B1 is associated with induction of beta-cell autoimmunity that portends type 1 diabetes. Diabetes 63, 446–455, doi: 10.2337/db13-0619 (2014). 23974921

[b7] Stene L. C. *et al.* Enterovirus infection and progression from islet autoimmunity to type 1 diabetes: the Diabetes and Autoimmunity Study in the Young (DAISY). Diabetes 59, 3174–3180, doi: 10.2337/db10-0866 (2010). 20858685 PMC2992780

[b8] Schmid S. *et al.* Reduced IL-4 associated antibody responses to vaccine in early pre-diabetes. Diabetologia 45, 677–685, doi: 10.1007/s00125-002-0816-7 (2002). 12107748

[b9] Giannopoulou E. Z. *et al.* Islet autoantibody phenotypes and incidence in children at increased risk for type 1 diabetes. Diabetologia 58, 2317–2323, doi: 10.1007/s00125-015-3672-y (2015). 26138334

[b10] Krischer J. P. *et al.* The 6 year incidence of diabetes-associated autoantibodies in genetically at-risk children: the TEDDY study. Diabetologia 58, 980–987, doi: 10.1007/s00125-015-3514-y (2015). 25660258 PMC4393776

[b11] Ziegler A. G. *et al.* Seroconversion to multiple islet autoantibodies and risk of progression to diabetes in children. Jama 309, 2473–2479, doi: 10.1001/jama.2013.6285 (2013). 23780460 PMC4878912

[b12] Oikarinen S. *et al.* Virus antibody survey in different European populations indicates risk association between coxsackievirus B1 and type 1 diabetes. Diabetes 63, 655–662, doi: 10.2337/db13-0620 (2014). 24009257

[b13] Yeung W. C., Rawlinson W. D. & Craig M. E. Enterovirus infection and type 1 diabetes mellitus: systematic review and meta-analysis of observational molecular studies. BMJ 342, d35, doi: 10.1136/bmj.d35 (2011). 21292721 PMC3033438

[b14] Kaufman D. L. *et al.* Autoimmunity to two forms of glutamate decarboxylase in insulin-dependent diabetes mellitus. The Journal of clinical investigation 89, 283–292, doi: 10.1172/JCI115573 (1992). 1370298 PMC442846

[b15] Lonnrot M. *et al.* Antibody cross-reactivity induced by the homologous regions in glutamic acid decarboxylase (GAD65) and 2C protein of coxsackievirus B4. Childhood Diabetes in Finland Study Group. Clinical and experimental immunology 104, 398–405 (1996). 9099922 10.1046/j.1365-2249.1996.60771.x PMC2200444

[b16] Muckelbauer J. K. *et al.* The structure of coxsackievirus B3 at 3.5 A resolution. Structure 3, 653–667 (1995). 8591043 10.1016/s0969-2126(01)00201-5

[b17] Roivainen M. *et al.* Several different enterovirus serotypes can be associated with prediabetic autoimmune episodes and onset of overt IDDM. Childhood Diabetes in Finland (DiMe) Study Group. Journal of medical virology 56, 74–78 (1998). 9700636 10.1002/(sici)1096-9071(199809)56:1<74::aid-jmv12>3.0.co;2-w

[b18] Dahlquist G. G., Ivarsson S., Lindberg B. & Forsgren M. Maternal enteroviral infection during pregnancy as a risk factor for childhood IDDM. A population-based case-control study. Diabetes 44, 408–413 (1995). 7698508 10.2337/diab.44.4.408

[b19] Hyoty H. *et al.* A prospective study of the role of coxsackie B and other enterovirus infections in the pathogenesis of IDDM. Childhood Diabetes in Finland (DiMe) Study Group. Diabetes 44, 652–657 (1995). 7789630 10.2337/diab.44.6.652

[b20] Viskari H. *et al.* Maternal enterovirus infection as a risk factor for type 1 diabetes in the exposed offspring. Diabetes care 35, 1328–1332, doi: 10.2337/dc11-2389 (2012). 22432113 PMC3357251

[b21] Viskari H. *et al.* Relationship between the incidence of type 1 diabetes and maternal enterovirus antibodies: time trends and geographical variation. Diabetologia 48, 1280–1287, doi: 10.1007/s00125-005-1780-9 (2005). 15902401

[b22] McPhee F., Zell R., Reimann B. Y., Hofschneider P. H. & Kandolf R. Characterization of the N-terminal part of the neutralizing antigenic site I of coxsackievirus B4 by mutation analysis of antigen chimeras. Virus research 34, 139–151 (1994). 7531922 10.1016/0168-1702(94)90096-5

[b23] Salur L. *et al.* Enterovirus infections in young infants: are children still protected by maternal antibodies? Human vaccines 7, 966–971, doi: 10.4161/hv.7.9.16082 (2011). 21860257

[b24] Bian X. *et al.* Immunoproteomic Profiling of Antiviral Antibodies in New-Onset Type 1 Diabetes Using Protein Arrays. Diabetes 65, 285–296, doi: 10.2337/db15-0179 (2016). 26450993 PMC4686945

[b25] Lipkin W. I. & Anthony S. J. Virus hunting. Virology 479–480, 194–199, doi: 10.1016/j.virol.2015.02.006 (2015). 25731958

[b26] Hou J., Said C., Franchi D., Dockstader P. & Chatterjee N. K. Antibodies to glutamic acid decarboxylase and P2-C peptides in sera from coxsackie virus B4-infected mice and IDDM patients. Diabetes 43, 1260–1266 (1994). 7523207 10.2337/diab.43.10.1260

[b27] Eugster A. *et al.* High diversity in the TCR repertoire of GAD65 autoantigen-specific human CD4+ T cells. J Immunol 194, 2531–2538, doi: 10.4049/jimmunol.1403031 (2015). 25681349

[b28] Eugster A. *et al.* Measuring T cell receptor and T cell gene expression diversity in antigen-responsive human CD4+ T cells. Journal of immunological methods 400–401, 13–22, doi: 10.1016/j.jim.2013.11.003 (2013). 24239865

[b29] Hummel S., Pfluger M., Hummel M., Bonifacio E. & Ziegler A. G. Primary dietary intervention study to reduce the risk of islet autoimmunity in children at increased risk for type 1 diabetes: the BABYDIET study. Diabetes care 34, 1301–1305, doi: 10.2337/dc10-2456 (2011). 21515839 PMC3114350

[b30] Thumer L. *et al.* German new onset diabetes in the young incident cohort study: DiMelli study design and first-year results. The review of diabetic studies: RDS 7, 202–208, doi: 10.1900/RDS.2010.7.202 (2010). 21409312 PMC3061610

[b31] Ziegler A. G., Meier-Stiegen F., Winkler C. & Bonifacio E. Prospective evaluation of risk factors for the development of islet autoimmunity and type 1 diabetes during puberty–TEENDIAB: study design. Pediatric diabetes 13, 419–424, doi: 10.1111/j.1399-5448.2011.00763.x (2012). 21446926

[b32] Ziegler A. G., Hummel M., Schenker M. & Bonifacio E. Autoantibody appearance and risk for development of childhood diabetes in offspring of parents with type 1 diabetes: the 2-year analysis of the German BABYDIAB Study. Diabetes 48, 460–468 (1999). 10078544 10.2337/diabetes.48.3.460

[b33] Lampasona V. *et al.* Diabetes antibody standardization program: first proficiency evaluation of assays for autoantibodies to zinc transporter 8. Clinical chemistry 57, 1693–1702, doi: 10.1373/clinchem.2011.170662 (2011). 21980171

[b34] Schlosser M., Mueller P. W., Torn C., Bonifacio E. & Bingley P. J. Diabetes Antibody Standardization Program: evaluation of assays for insulin autoantibodies. Diabetologia 53, 2611–2620, doi: 10.1007/s00125-010-1915-5 (2010). 20871974

[b35] Torn C., Mueller P. W., Schlosser M., Bonifacio E. & Bingley P. J. Diabetes Antibody Standardization Program: evaluation of assays for autoantibodies to glutamic acid decarboxylase and islet antigen-2. Diabetologia 51, 846–852, doi: 10.1007/s00125-008-0967-2 (2008). 18373080

[b36] Catani M. *et al.* Isolation of human monoclonal autoantibodies derived from pancreatic lymph node and peripheral blood B cells of islet autoantibody-positive patients. Diabetologia 59, 294–298, doi: 10.1007/s00125-015-3792-4 (2016). 26493188

[b37] Berthold M. R. *et al.* In Data Analysis, Machine Learning and Applications. (eds Christine Preisach, Hans Burkhardt, Lars Schmidt-Thieme & Reinhold Decker) 319–326 (Springer Berlin Heidelberg, 2008).

[b38] Lefranc M. P. *et al.* IMGT, the international ImMunoGeneTics information system. Nucleic acids research 37, D1006–D1012, doi: 10.1093/nar/gkn838 (2009). 18978023 PMC2686541

