# Supplementary material for: Incomplete immune response to coxsackie B viruses associates with early autoimmunity against insulin
Source: Sci Rep. 2016 Sep 8;6:32899. doi: 10.1038/srep32899 (PMC5015062; doi:10.1038/srep32899)
Supplement: Supplementary Information [file srep32899-s1.pdf]

Incomplete immune response to Coxsackie B viruses associates with early autoimmunity against insulin

Michelle P. Ashton[1], Anne Eugster[1], Denise Walther[1], Natalie Daehling[1], Stephanie Riethausen[2], Denise Kuehn[1], Karin Klingel[3], Andreas Beyerlein[2], Stephanie Zillmer[2], Anette-Gabriele Ziegler[2,4,5], Ezio Bonifacio[1,4,5,*]

[1]DFG-Center for Regenerative Therapies Dresden, Dresden, Germany

[2]Institute of Diabetes Research, Helmholtz Zentrum München and Forschergruppe Diabetes, Klinikum rechts der Isar, Technische Universität München, Neuherberg, Germany

[3]Department of Molecular Pathology, University Hospital Tübingen, Tübingen, Germany

[4]Forschergruppe Diabetes e.V., Neuherberg, Germany

[5]German Center for Diabetes Research (DZD), Neuherberg, Germany

*ezio.bonifacio@crt-dresden.de

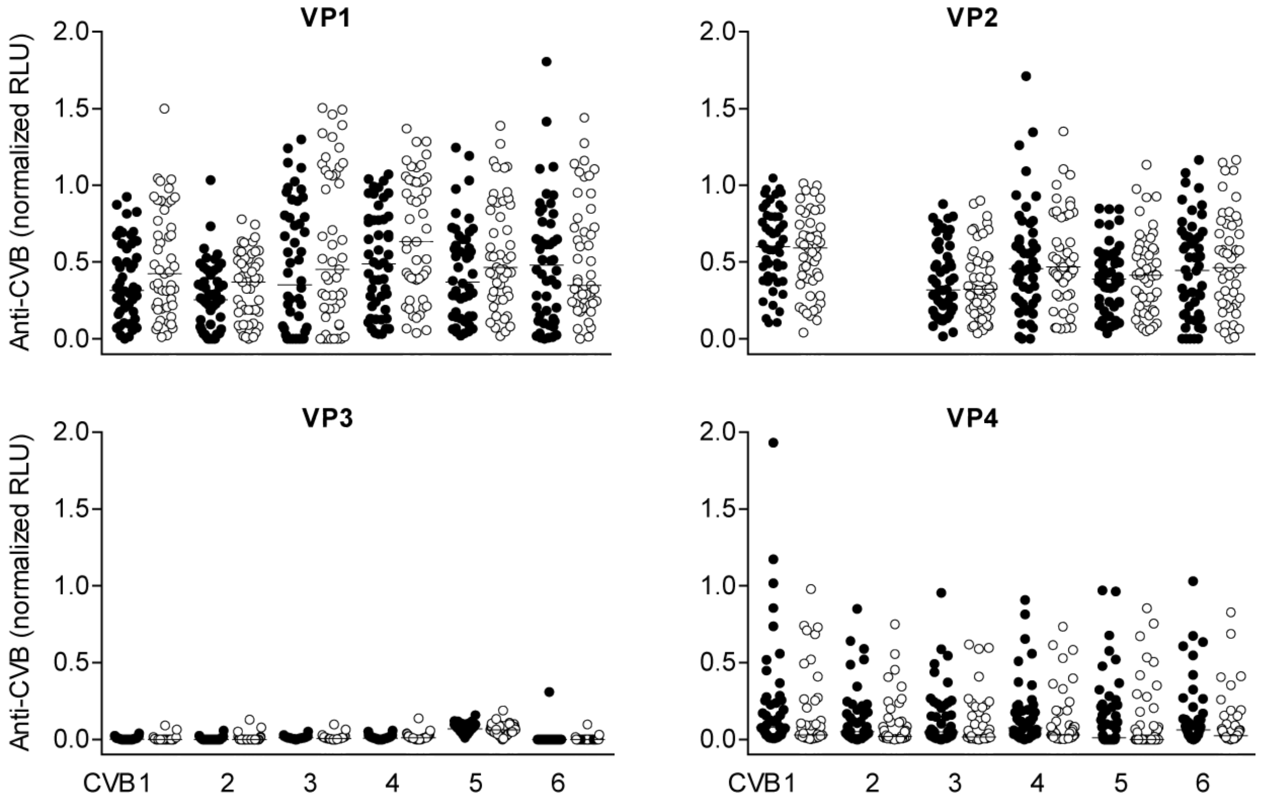

Supplementary Figure 1: Anti-VP1, anti-VP2, and anti-VP4 CVB antibodies can be detected in serum

LIPS measurement of anti-VP1, anti-VP2, anti-VP3 and anti-VP4 antibodies in sera from children and adolescents with recent onset of type 1 diabetes (open circles; n=78) and healthy controls (filled circles; n=78). Each point is an individual and the line indicates the median.

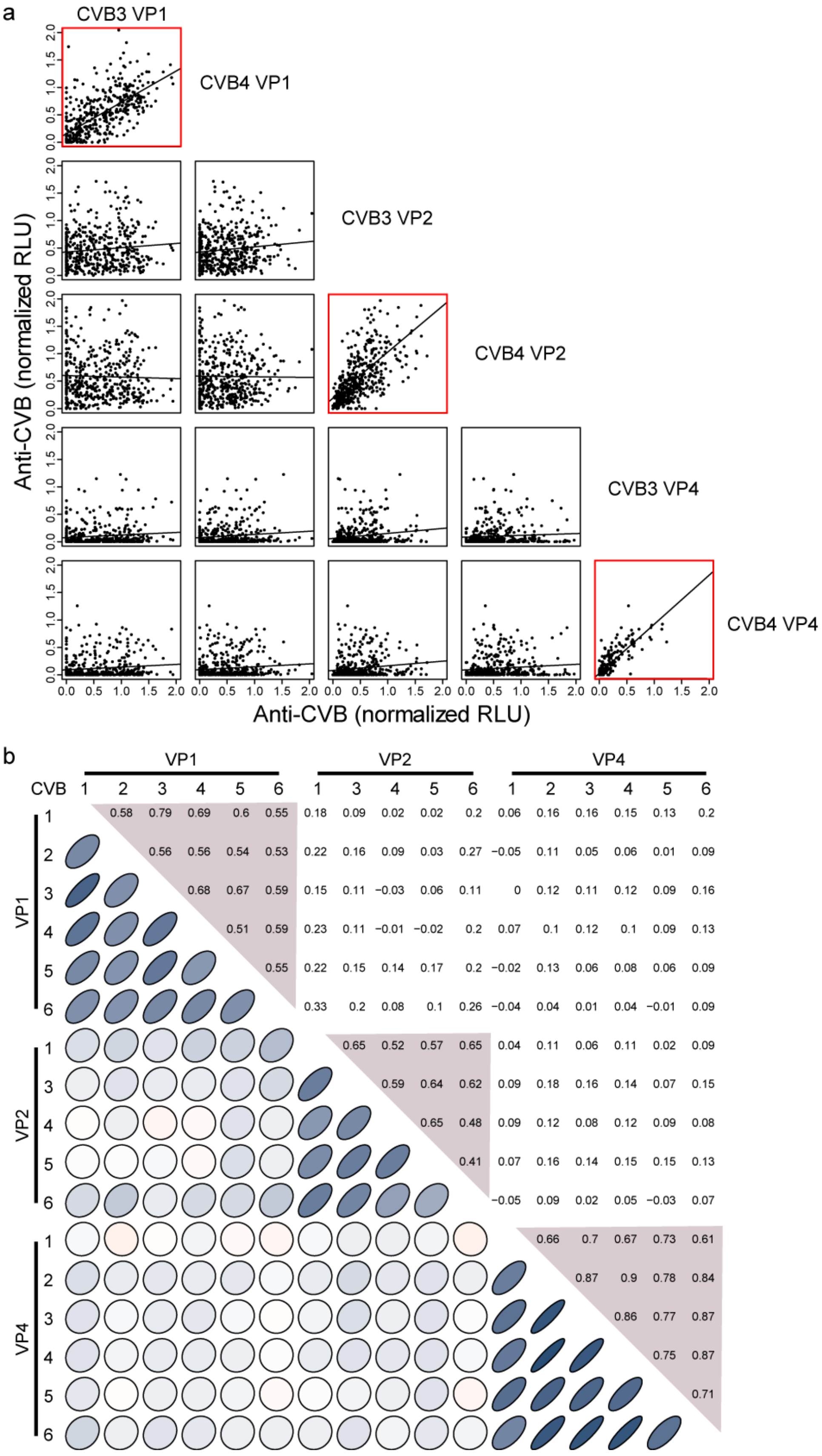
a
CVB3 VP1
CVB4 VP1
CVB3 VP2
CVB4 VP2
CVB3 VP4
CVB4 VP4
0.0 0.5 1.0 1.5 2.0
Anti-CVB (normalized RLU)
Anti-CVB (normalized RLU)
b
VP1
VP2
VP4
CVB 1 2 3 4 5 6 1 3 4 5 6 1 2 3 4 5 6
1 0.58 0.79 0.69 0.6 0.55 0.18 0.09 0.02 0.02 0.2 0.06 0.16 0.16 0.15 0.13 0.2
2 0.56 0.56 0.54 0.53 0.22 0.16 0.09 0.03 0.27 −0.05 0.11 0.05 0.06 0.01 0.09
3 0.68 0.67 0.59 0.15 0.11 −0.03 0.06 0.11 0 0.12 0.11 0.12 0.09 0.16
4 0.51 0.59 0.23 0.11 −0.01 −0.02 0.2 0.07 0.1 0.12 0.1 0.09 0.13
5 0.55 0.22 0.15 0.14 0.17 0.2 −0.02 0.13 0.06 0.08 0.06 0.09
6 0.33 0.2 0.08 0.1 0.26 −0.04 0.04 0.01 0.04 −0.01 0.09
1 0.65 0.52 0.57 0.65 0.04 0.11 0.06 0.11 0.02 0.09
3 0.59 0.64 0.62 0.09 0.18 0.16 0.14 0.07 0.15
4 0.65 0.48 0.09 0.12 0.08 0.12 0.09 0.08
5 0.41 0.07 0.16 0.14 0.15 0.15 0.13
6 −0.05 0.09 0.02 0.05 −0.03 0.07
1 0.66 0.7 0.67 0.73 0.61
2 0.87 0.9 0.78 0.84
3 0.86 0.77 0.87
4 0.75 0.87
5 0.71
6

Supplementary Figure 2: Correlations between antibodies to viral capsid antigens from CVB1- 6.

(**a**) Scatter plot matrix of anti-CVB3 and anti-CVB4 antibody titers in all sera analyzed in this study. Each dot represents a sample and the line represents the linear regression. Plots with the same VP (but different serotype) plotted on both the x and y axes are highlighted with a red border. (**b**) A matrix summarizing the Pearson correlation between antibody titers for each CVB antigen. The upper right panel lists the correlation co-efficient for each comparison and values greater than 0.4 are shaded. The bottom left panel shows ellipses representative of the magnitude of the correlation. Blue ellipses with a positive slope, indicate a positive correlation and red ellipses with a negative slope indicate negative correlations, where a greater intensity in color indicates a correlation-coefficient closer to 1 (blue) or -1 (red).

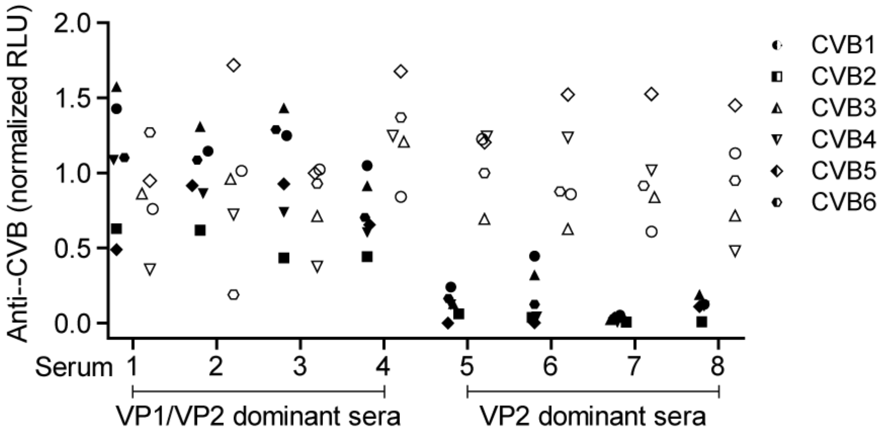

Supplementary Figure 3: Sera selected for analysis by plaque neutralization test.

Anti-VP1 (filled symbols) and anti-VP2 (open symbols) antibody titers of sera selected to be analyzed by the plaque neutralization test as determined by the LIPS assay. Sera 1-4 have strong anti-VP1 and anti-VP2 antibody titers and sera 5-8 have strong anti-VP2 antibody titers.

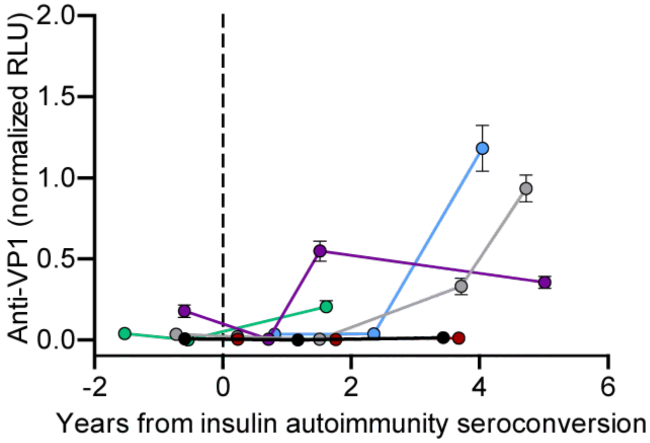

Supplementary Figure 4: Anti-CVB antibodies in follow-up samples from children with early insulin autoimmunity.

Anti-VP1 antibodies (y axis) in longitudinal serum samples from children who developed beta cell autoantibody seroconversion before age 3 years with IAA as the first detected autoantibodies. Titers are shown relative to the time of insulin autoantibody seroconversion (x axis). Each symbol and line represents a different child, each point represents the mean RLU for VP1 of all six CVB serotypes measured at a given time point, and error bars represent s.e.m.

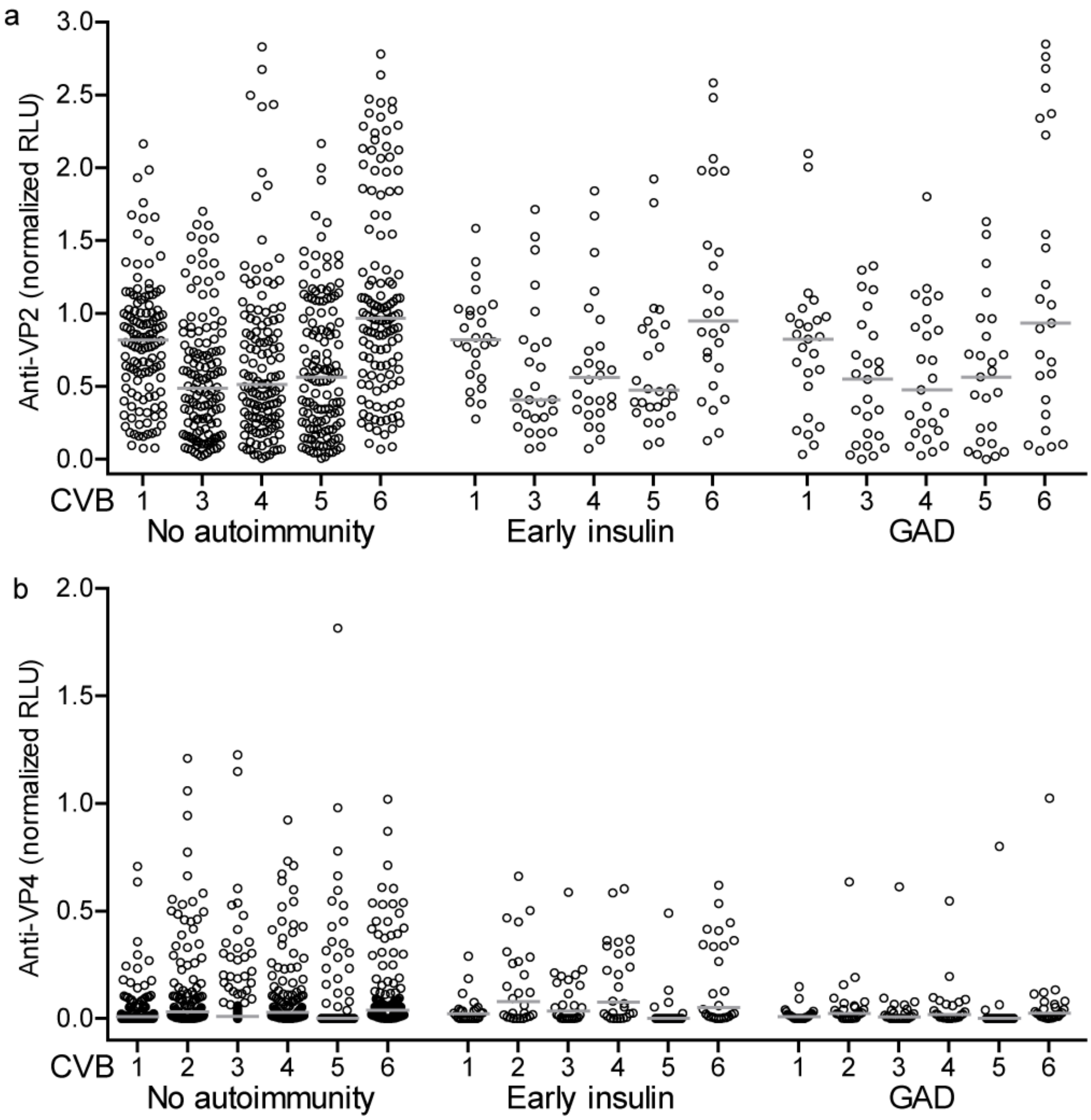

Supplementary Figure 5: Anti-VP2 and anti-VP4 CVB antibodies in newborns are not associated with seroconversion.

Anti-VP2 (**a**) and anti-VP4 (**b**) antibodies in cord blood serum of BABYDIAB study participants stratified as beta cell autoantibody negative throughout follow-up (no autoimmunity), beta cell autoantibody seroconversion before age 3 years with IAA as the first detected autoantibodies (early insulin), and beta cell autoantibody seroconversion with GAD as the first detected autoantibody target (GAD). Each point is an individual and the horizontal bar indicates the median. No significant differences based on comparison with early insulin autoimmunity group for the corresponding CVB serotype using the Mann Whitney U test.

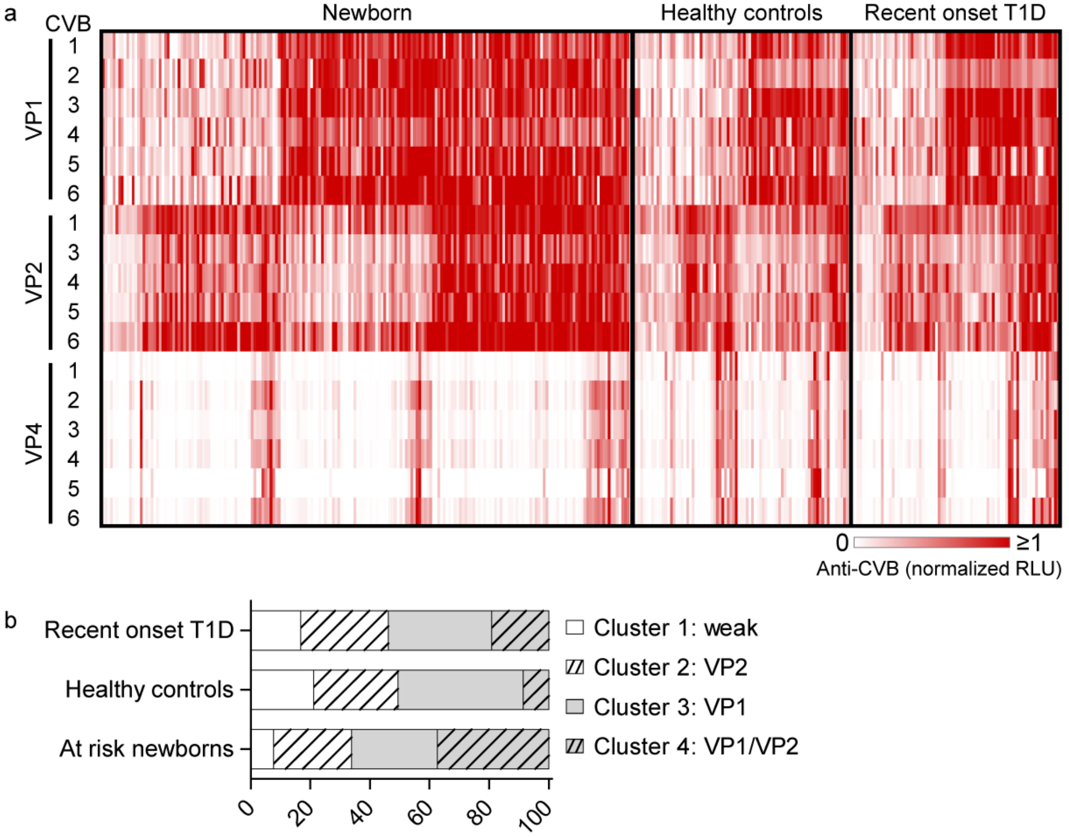

Supplementary Figure 6: Maternal anti-CVB antibody profiles in newborns differ from those observed in adolescents.

The LIPS assay was used to detect antibodies to viral capsid protein (VP) antigens of CVB1-6 in cord blood serum (Newborn), healthy control children and adolescents (Healthy controls), and children and adolescents with recent onset type 1 diabetes (Recent onset T1D). (**a**) Antibody titers against VPs of CBV1-6 are represented as a heat map where each vertical line is a sample and the intensity of the color corresponds to the RLU measured by the LIPS assay. Data is sorted based on the clusters that were identified as described in Fig. 1. (**b**) The proportion of sera within the 4 clusters defined in Fig. 1 is represented by a stacked bar chart for each cohort. A higher proportion of samples from newborns were observed in cluster 4 (p>0.001 Healthy controls; p=0.004 Recent onset T1D) and a lower proportion of samples from newborns were observed in cluster 1 (p=0.002 Healthy controls; p=0.027 Recent onset T1D) as compared to the children/adolescent groups using the Z-score test statistic and two-tailed test.

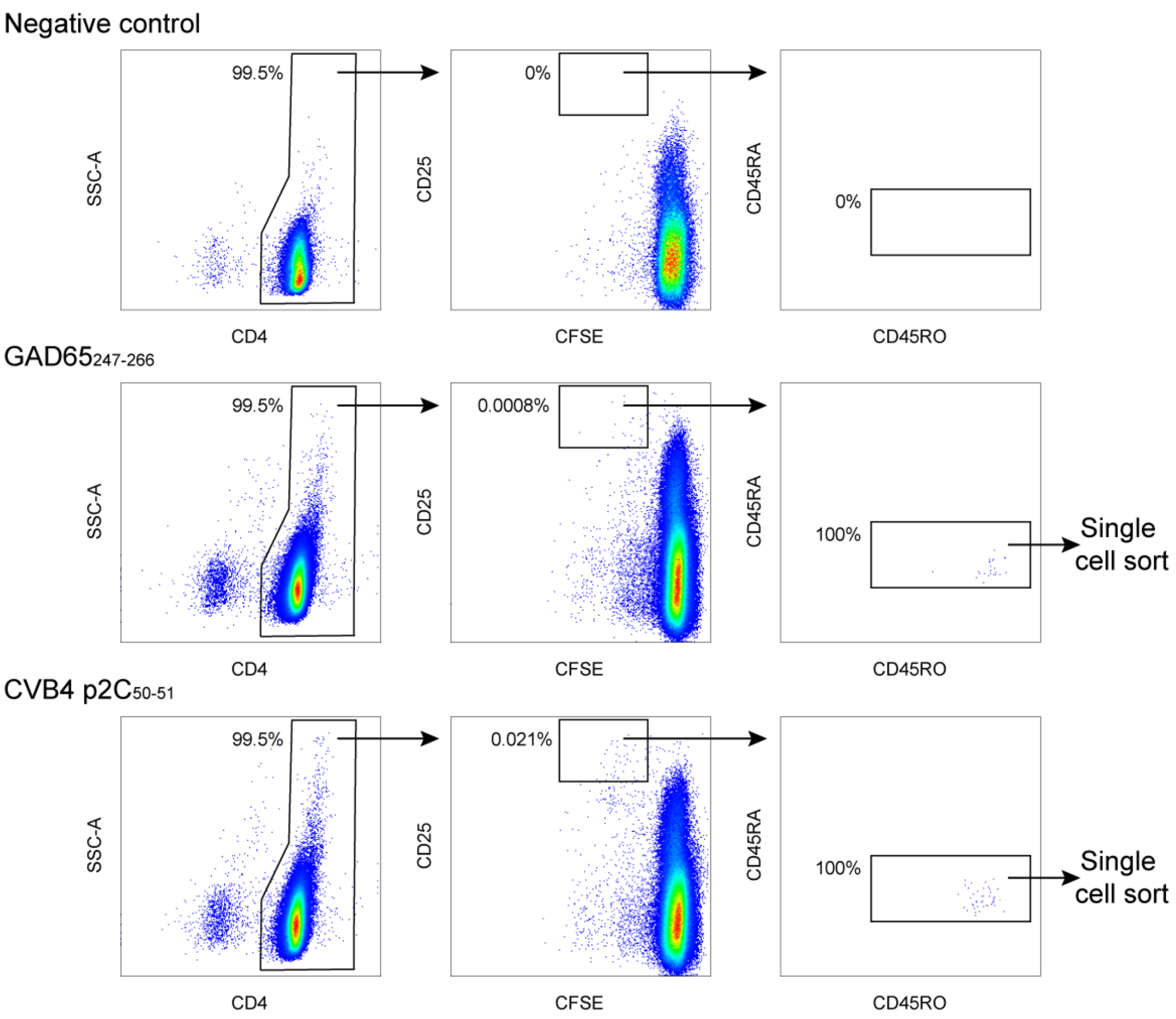

Supplementary Figure 7: Gating strategy for isolation of peptide responsive CD45RO$^+$ T cells.

CFSE-labeled CD45RO$^+$ T cells cultured with CVB4 p2C$_{30-51}$ or GAD65$_{247-266}$ peptide loaded monocyte-derived dendritic cells for 5 days. Antigen-responsive memory T cells were identified by flow cytometry as 7AAD$^-$CD4$^+$CD25^{hi}CFSE^{lo}CD45RO$^+$CD45RA$^-$.

Supplementary table 1: Anti-VP1 and anti-VP4 antibody titers increase with age.

			Group 1 (mean ± s.e.m)			Group 2 (mean ± s.e.m)			p-value*
**Age (years)**			2.783	±	0.039	12.01	±	0.120	
**Relative light units (normalized)**	**VP1**	CVB1	0.272	±	0.038	0.557	±	0.031	>0.0001
		CVB2	0.296	±	0.062	0.339	±	0.019	>0.0001
		CVB3	0.281	±	0.044	0.642	±	0.041	>0.0001
		CVB4	0.139	±	0.025	0.584	±	0.029	>0.0001
		CVB5	0.312	±	0.055	0.503	±	0.029	>0.0001
		CVB6	0.219	±	0.031	0.623	±	0.042	>0.0001
	**VP2**	CVB1	0.507	±	0.033	0.588	±	0.021	0.0552
		CVB3	0.448	±	0.028	0.390	±	0.019	0.0844
		CVB4	0.662	±	0.064	0.471	±	0.027	0.1022
		CVB5	0.816	±	0.068	0.519	±	0.031	0.0010
		CVB6	0.551	±	0.037	0.526	±	0.027	0.5364
	**VP4**	CVB1	0.067	±	0.021	0.162	±	0.025	>0.0001
		CVB2	0.093	±	0.023	0.124	±	0.015	>0.0001
		CVB3	0.100	±	0.020	0.133	±	0.017	0.0481
		CVB4	0.083	±	0.020	0.148	±	0.017	>0.0001
		CVB5	0.096	±	0.034	0.141	±	0.027	0.0002
		CVB6	0.081	±	0.017	0.171	±	0.021	0.0002

*p-values calculated using Mann Whitney U Test

Supplementary table 2: Paired TCR sequences not shared between CVB4 p2C peptide and GAD65 peptide responsive $CD4^+CD45RO^+$ T cells.

Subject	Antigen	# cells	TRAV	TRAJ	JunctionA	TRBV	TRBJ	TRBD	JunctionB
BC928	CVB4 p2C$_{30-51}$	1	36/DV7*02	42*01	CAVEA#YGGSQGNLIF	20-1*01	2-7*01	2*02	CSARDLRELEQYF
		1	36/DV7*02	42*01	CAVEANMEXAK#NLIF	20-1*01	2-7*01	2*02	CSARDLRELEQYF
		1	9-2*01	30*01	CALT#NRDDKIIF	28*01	2-3*01	1*01	CASSLAGQTPDTQ#F
		1	9-2*01	30*01	CALTGTEMTRSS#	9*01	2-3*01	1*01	CASSLAGQTPDTQ#F
		1	9-2*01	34*01	CVVSVGNTDKLIF	10-3*01	2-3*01	1*01	CASSLAGQTPDTQ#F
		1	9-2*01	29*01	CAVGARGSGNTPLVF	30*01	2-3*01	1*01	CASSLAGQTPDTQ#F
		1	26-1*01	42*01	CIVRG#YGGSQGNLIF	10-3*01	2-1*01	1*01	CASSIGGTFGPYNEQFF
		1	22*01	28*01	CAVSGAGSYQLTF	5-1*01	2-5*01	2*01	CASSLSTSVAQETQYF
		1	8-2*01	34*01	CVVSVGNTDKLIF	28*01	1-5*01	1*01	VPSVPRQE#QPQHF
		1	26-1*01	58*01	CIVSKETSGSRLTF	6-1*01	2-1*01	1*01	CATRSTGNIYNEQFF
		1	26-1*01	33*01	CIVRFGP#MDSNYQLIW	6-1*01	2-3*01	2*01	CRARVSATSTDTQ#F
		1	36/DV7*02	54*01	CAVEAVIQGAQKLVF	2*01	2-3*01	2*02	CASSQDGASGRAYTQYF
		1	36/DV7*02	42*01	CAVEXNMEEAXX#LIF	27*01	2-7*01	2*02	CRARDLRELXEQYF
		1	9-2*01	21*01	CALLSYNFNKFYF	28*01	2-1*01	2*01	CASXKRD*RGGLQ*A#FF
		1	23/DV6*01	13*02	CAASRTSGGYQKVTF	5-4*01	1-6*02	1*01	CASRGQGAASPLHF
		1	17*01	56*01	CATDYTGANSKLTF	4-2*01	2-1*01	2*01	CSAQXTPRPVSS#F
		1	23/DV6*01	29*01	CAARYSGNTPLVF	5-6*01	2-7*01	1*01	CRANGAR#EQYF
		1	8-6*01	49*01	CAVILL#NQFYF	14*01	2-1*01	1*01	FTSSAARYEQFF
		1	36/DV7*02	40*01	CAVEG#YKYIF	3-1*01	2-3*01	1*01	CRXGDRGTDTQ#F
		1	9-2*01	52*01	CALSQNAGGTSYGKLTF	19*01	2-5*01	1*01	CASSSRDRPTQYF
		1	9-2*01	30*01	CALTGTEMTSSS#	28*01	2-3*01	1*01	CASSLAGRPQIR##F
		1	41*01	49*01	CADHTNTGNQFYF	2*01	2-5*01	2*01	CASSALSSVKKETQYF
		1	17*01	23*01	CATLNVGNQGGKLIF	7-6*01	2-6*01	1*01	CASXEGQGLGGWVNXLTF
		1	17*01	23*01	CATLNXGNQGGKLIF	5-5*01	2-6*01	1*01	CAXXEGRGWALGPTAX#F
		1	8-6*02	52*01	CAVSYAGGTSYGKLTF	6-6*01	1-5*01	1*01	CASSGXGGQPQHF
	GAD65$_{247-266}$	1	26-1*01	26*01	CIVRVSHN*G#RDNYGQNFVF	10-3*01	2-5*01	1*01	CAS*GQGQETQNF
		1	29/DV5*01	33*01	CAASDGSNYQLIW	27*01	2-7*01	1*01	CASSLPRTGKDEQYF
		1	29/DV5*01	6*01	CAASGSGGSYIPTF	27*01	2-3*01	2*01	CARQGD*RKXDTQ#F
		1	6*03 []	4*01	CALDPGGYNKLFF	7-9*01	2-1*01	2*01	CSARTSGGYNEQFF
		1	2*01	39*01	CAVEDHILLXNLLPF	5-1*01	2-1*01	2*01	CASSLVFTGSGYNEQFF
		1	8-6*02	50*01	CAVRRASYDKVIF	7-9*01	2-2*01	2*02	CASKSSGRLTGELFF
		1	3*01	6*01	CAVRVLGGSYIPTF	5-1*01	2-1*01	2*01	CTGTREYNEQFF
		1	8-6*01	31*01	CAVSEGX#RLMF	28*01	2-2*01	2*01	CASTPRGRETGGLFF
		1	8-2*01	56*01	CAVSGAA*GLX#NSKLTF	29-1*01	2-7*01	2*01	CASSERGGS#EQYF
		1	26-1*01	42*01	CIVLRL#GSQGNLIF	28*01	2-2*01	2*02	CASRGLAGATGELFF
		1	26-1*01	33*01	CIVRFGPKWI#NYQLIW	2*01	2-3*01	1*01	CSARVSATSTDTQYF
		1	26-1*01	38*01	CIVRG*CWQQPYAEL#	6-5*01	2-3*01	2*01	CRAKLGLEATDTQ#F
		1	26-1*01	38*01	CIVRG*CWQQPYPEP#	20-1*01	2-3*01	2*01	CSARLGLEATDTQYF
		1	8-4*03	49*01	CVASLNTGNQFYF	20-1*03	2-5*01	1*01	CASSLDRVQETQYF
		1	29/DV5*01	30*01	VQQE#DDKIIF	20-1*01	2-1*01	2*01	CASSVGLAKTIGEQFF
		1	25*01	39*01	CAALVGWAGNMLTF	5-6*01	2-7*01	2*01	CVPAIIRGGGXYEEYF
		1	2*01	29*01	CAASRNSGNTPLVF	1*01	2-3*01	1*01	CARSWEDTQXF
B858	GAD65$_{247-266}$	5	8-3*02	20*01	CAVGSNDYKLSF	20-1*04	1-2*01	1*01	CSAPSGPP#NYGYTF
		2	41*01	42*01	CAVMNYGGSQGNLIF	11-1*01	2-2*01	2*01	CASDRGGKPGGVVF
D161	CVB4 p2C$_{30-51}$	2	29/DV5*01	31*01	CAAFRNNNARLMF	5-5*01	1-3*01	1*01	CASSLFSSGNTIYF
		1	36/DV7*01	44*01	CAVEAAGTASKLTF	30*01	1-1*01	2*01	CAWGHSGGPTNTEAFF
